# Quantitative cardiovascular magnetic resonance for molecular imaging

**DOI:** 10.1186/1532-429X-12-62

**Published:** 2010-11-03

**Authors:** Patrick M Winter, Shelton D Caruthers, Gregory M Lanza, Samuel A Wickline

**Affiliations:** 1 Cincinnati Children's Hospital, Department of Radiology, 3333 Burnet Ave., ML 5033, Cincinnati, OH, 45229, USA; 2 Washington University, C-TRAIN Labs, 660 S. Euclid Ave., Campus Box 8215, St. Louis, MO, 63110, USA

## Abstract

Cardiovascular magnetic resonance (CMR) molecular imaging aims to identify and map the expression of important biomarkers on a cellular scale utilizing contrast agents that are specifically targeted to the biochemical signatures of disease and are capable of generating sufficient image contrast. In some cases, the contrast agents may be designed to carry a drug payload or to be sensitive to important physiological factors, such as pH, temperature or oxygenation. In this review, examples will be presented that utilize a number of different molecular imaging quantification techniques, including measuring signal changes, calculating the area of contrast enhancement, mapping relaxation time changes or direct detection of contrast agents through multi-nuclear imaging or spectroscopy. The clinical application of CMR molecular imaging could offer far reaching benefits to patient populations, including early detection of therapeutic response, localizing ruptured atherosclerotic plaques, stratifying patients based on biochemical disease markers, tissue-specific drug delivery, confirmation and quantification of end-organ drug uptake, and noninvasive monitoring of disease recurrence. Eventually, such agents may play a leading role in reducing the human burden of cardiovascular disease, by providing early diagnosis, noninvasive monitoring and effective therapy with reduced side effects.

## Introduction

While cardiovascular magnetic resonance (CMR) occupies a prominent role in anatomical and functional examinations of the heart and the major vessels [1-3], molecular imaging aims to identify and map the expression of important biomarkers on a cellular scale. Typical CMR techniques lack sufficient resolution and sensitivity to directly detect these molecular signatures due to their very low concentrations in vivo. Important metabolites and biomolecules, such as glucose, adenosine triphosphate (ATP), lactate and others, can be detected through MR spectroscopy, but in vivo mapping of these compounds is not widely available for routine biomedical applications due to the need for specialized instrumentation or very high concentrations. Instead, CMR molecular imaging relies on the development of a new class of contrast agents, which are specifically targeted to the biomarker of interest and are capable of amplifying the signal enhancement to generate sufficient image contrast. These contrast agents often take the form of nanoparticle constructs, which offer a large surface area for the incorporation of multiple binding ligands (to improve targeting efficacy) and multiple paramagnetic chelates (to amplify the signal enhancement). In some cases, the nanoparticle may also serve as a drug delivery agent, providing diagnostic and therapeutic information via noninvasive MRI. Some CMR contrast agents are not designed to bind to specific biomarkers, but instead are sensitive to important physiological factors, such as pH, temperature or oxygenation.

Quantification is an important feature of many CMR applications, such as measuring ventricular volumes or blood velocity. In the arena of molecular imaging, quantitation could allow stratification of patient risk levels, serial monitoring of therapeutic response or noninvasive mapping of drug concentrations at the target tissue. Quantification of CMR data can take various forms. CMR molecular imaging data can be quantified based on the amount of signal change, the area of tissue displaying contrast enhancement, calculations of relaxation time changes or direct detection of contrast agents through multi-nuclear imaging or spectroscopy.

A number of other medical imaging modalities are capable of quantitative molecular imaging, including PET, SPECT and optical imaging [4-6]. Like CMR, these modalities typically rely on specifically designed contrast agents that bind to the molecular biomarker of interest. For PET and SPECT imaging, the contrast agents consist of a radioactive element for detection. In optical imaging, the contrast agent will utilize fluorescence or bioluminescence for mapping expression of cellular receptors. CMR, however, has certain important advantages over these other modalities for molecular imaging applications. CMR offers higher spatial and temporal resolution than PET or SPECT and provides greater tissue penetration than optical imaging. This allows CMR to combine anatomical and/or functional information with molecular imaging, much like the multimodality benefits of PET/CT. In addition, CMR may be better suited for serial tracking of disease progression or therapeutic response than the nuclear imaging methods due to concerns of cumulative radiation dose.

## Overview of CMR Molecular Imaging

CMR molecular imaging has been applied to a wide range of diseases and utilized a variety of imaging techniques and contrast agents. Typically, molecular imaging contrast agents are based on either paramagnetic gadolium chelates or superparamagnetic iron oxide particles [7]. Gadolinium agents have been grafted onto targeting molecules, such as antibodies or peptides, to directly bind to important biomarkers of disease, such as fibrin. One of the hallmarks of a ruptured atherosclerotic plaque is the accumulation of thrombi in the vessel lumen. Identifying and localizing ruptured plaques would have an enormous benefit in the clinical diagnosis and treatment of myocardial infarction and stroke. Phage display has been used to develop a novel peptide that specifically binds to fibrin. This ligand was modified with four Gd-DTPA chelates per peptide to generate signal enhancement on CMR images. The abundance of fibrin in clots allows imaging thrombi in the left atrium, pulmonary arteries, and coronary arteries in animal models [8-14] and in clinical patients [15].

Another biomarker of clinical importance that has been pursued with paramagnetic gadolinium contrast agents is the uptake of lipoproteins within atherosclerotic plaques. Recombinant paramagnetic HDL particles were used to image atherosclerotic regions in apoE-deficient mice. The HDL particle system is formulated with 15-20 Gd-DTPA chelates per particle to provide ample CMR signal enhancement [16] and could be coupled to a macrophage scavenger receptor antibody for specific imaging of macrophage rich plaques [17]. Another contrast agent that selectively accumulates in fatty deposits of atherosclerotic plaques is gadofluorine. This gadolinum chelate contains fluorinated side chains that are hydrophobic. The side chains cause gadofluorine to form nanometer sized micelles when dispersed in water. These small lipophilic particles have been shown to preferentially accumulate in the lipid-rich areas of vascular plaques in cholesterol-fed rabbits [18].

The therapeutic uses of stem cells have become a rapidly growing field of research. A number of new CMR contrast agents and methods have been developed with the aim of tracking stem cell delivery, migration, viability and fate. Iron oxide contrast agents have dominated stem cell tracking studies [19-22]. In most cases, cultured stem cells are labeled in vitro with iron oxide particles. Cell labeling is often accomplished with exogenous transfection agents that induce uptake of the particles by endocytosis. The labeled cells are subsequently injected into the tissue of interest and detected in the CMR image based on the loss of signal due to enhancement of T2* relaxation. The use of iron oxide particles provides abundant image contrast, which allows detection of even a single labeled cell [23].

In addition to in vitro labeling of stem cells, iron oxide particles have been utilized for in vivo imaging of macrophages associated with atherosclerotic plaques. Activated macrophages spontaneously uptake particulate contrast agents by phagocytosis in animal models [24-27] and clinical atherosclerosis [28,29]. One limitation of MRI after systemic injection of iron oxide contrast agents is that they stay in the blood pool for a long time, requiring a delay between injection and imaging on the order of hours to days. Clinical imaging of carotid plaques required 24 hours between injection and imaging, which resulted in a 24% decrease in the image intensity of the plaques [28]. The negative image contrast associated with iron oxide particles has been considered a drawback for these methods. As an alternative approach, new pulse sequences and image processing routines have been developed to generate positive contrast in the CMR images [30-37]. For example, a method called "Inversion-recovery with ON-resonant water suppression", abbreviated as IRON, has been demonstrated to generate bright image contrast that was correlated with the number of labeled stem cells [36].

Iron oxide particles have also been formulated for ligand directed targeting of cellular biomarkers. These agents have been specifically targeted to holo-transferrin [38], E-selectin [39], a peptide sequence from the transactivator protein (Tat) of HIV-1 [40-42], annexin V [43] and VCAM-1 [44]. Specific targeting of iron oxide particles allows MRI detection of very sparse cellular receptors. These particles have very high relaxivities, causing large decreases in the T2* relaxation times of tissues expressing the biomarker of interest. Typically, the specificity of the molecular targeting approach is confirmed by histological staining of the targeted epitope combined with microscopic localization of the iron oxide with Prussian blue staining. However, based on the delays normally required between contrast agent injection and imaging, the nonspecific uptake of particles in macrophages may overshadow accumulation at the target site.

Another novel application of iron oxide particles is the construction of magnetic relaxation switches that change their MR relaxation properties based on interactions with biomolecules associated with various disease states [45]. For example, particles have been formulated with numerous copies of high-affinity ligands that bind myeloperoxidase, an enzyme produced by inflammatory cells that is associated with plaque vulnerability [46]. In another cardiovascular application, iron oxide agents conjugated to a2AP peptides have been used to detect activated Factor 13, an enzyme that crosslinks fibrin and stabilizes clots [47].

## Quantification via Signal Enhancement

One of the most convenient methods for quantifying paramagnetic contrast agent uptake is by simply calculating the change in the image intensity. Some studies have demonstrated that the CMR signal is linearly related to the contrast agent concentration within the typical in vivo range [48,49]. However, this method depends strongly on the imaging parameters and requires careful calibration. In addition to actually reporting contrast agent concentrations, CMR signal enhancement can be used to quantify the physical size of abnormal tissue, such as the volume of infarcted myocardium or the extent of abnormal vessel permeability.

### Quantification of Pharmacokinetics

CMR signal enhancement was used to quantify the pharmacokinetics of a molecular imaging contrast agent in both the blood pool as well as the target tissue in an atherosclerotic rabbit model [49]. Paramagnetic nanoparticles that bind to the α_ν_β_3_-integrin, a biomarker of angiogenesis, were produced by incorporating a highly specific targeting ligand onto the particle surface. Atherosclerotic rabbits were injected with either α_ν_β_3_-targeted or nontargeted parmagnetic nanoparticles at a dose of 1 mL/kg (0.0046 mmol Gd^3+^/kg). As a point of reference, the standard dose of conventional gadolinium agents for clinical CMR scans is 0.1 mmol Gd^3+^/kg, which is 20-fold higher than the dose delivered with paramagnetic nanoparticles. Blood sampling was performed to determine the bulk pharmacokinetic behavior of the nanoparticles and T1-weighted CMR images of the descending aorta were collected over 24 hours to ascertain binding to the angiogenic microvasculature in the vessel wall. CMR was performed on a clinical 1.5T scanner with a cross-sectional, multislice, T1-weighted, turbo spin echo, fat-suppressed, black-blood imaging sequence.

The experimental data was fit to pharmacokinetic models in two steps. First, a standard two compartment pharmacokinetic model was used to describe only the blood concentrations without consideration of the imaging data. Then, a three-compartment pharmacokinetic model was developed to describe the in vivo behavior of the nanoparticles in both the blood pool as well as the aortic wall using the imaging data to represent the nanoparticle concentrations in the vessel wall. Compartments 1 and 2 represent the bulk distribution of nanoparticles throughout the blood stream. The third compartment consists of the vasa vasorum of the aortic wall where the nanoparticles can specifically bind to the α_ν_β_3_-integrin. The constants that describe the transfer into and out of this compartment, k_13 _and k_31_, are lumped parameters that are used to describe both passive transfer and active binding.

Blood samples were spiked with nanoparticles to yield gadolinium concentrations ranging from 0 to 45 μM. These calibration samples were imaged using the in vivo pulse sequence to determine the relationship between nanoparticle concentration and signal enhancement. The image enhancement varied linearly with gadolinium concentration at these low concentrations, in agreement with mathematical simulations of the turbo spin echo CMR sequence. T1 mapping was also performed using a mixed spin echo inversion recovery pulse sequence [50] to measure the longitudinal relaxivity of the nanoparticles in blood, 12.7 (s*mM)^-1^. Blood sampling after IV injection of nanoparticles demonstrated that the gadolinium concentration decayed bi-exponentially over time, as is typically observed with these nanoparticle agents [51]. Fitting the experimental data to the two-compartment pharmacokinetic model did not reveal any significant differences between the rabbits treated with targeted versus nontargeted nanoparticles. The half-life for the distribution phase was 20.2 minutes, while the elimination half-life was 11.9 hours.

**Figure 1:**
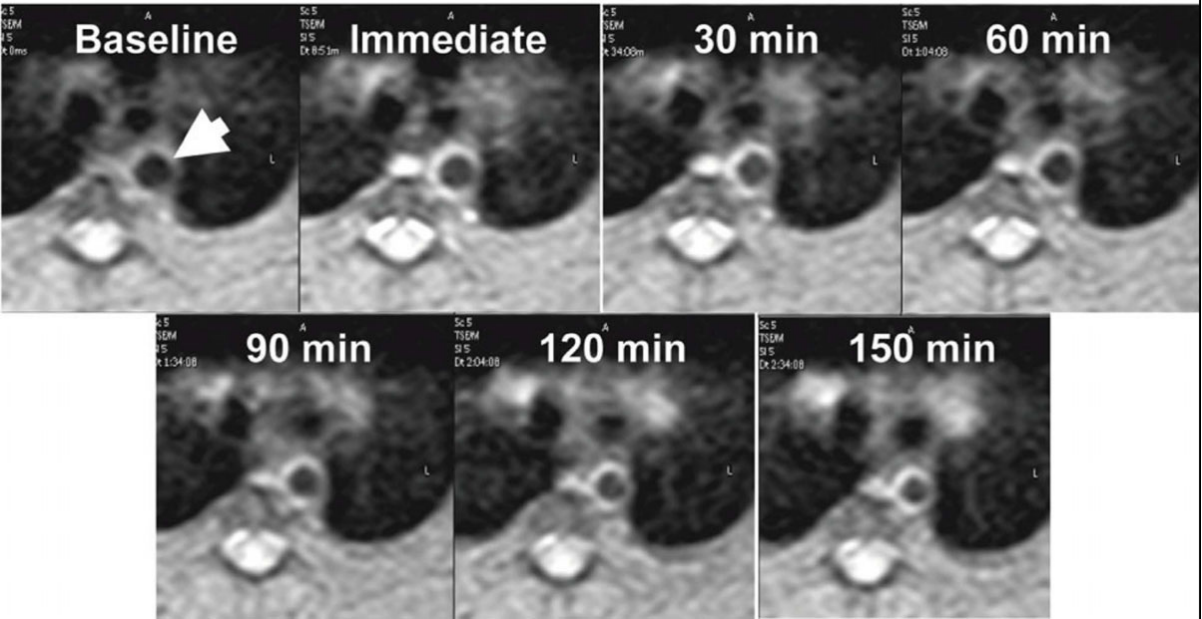
**In vivo CMR molecular imaging of angiogenesis in atherosclerosis**. Serial imaging of the aortic wall (arrow) of an atherosclerotic rabbit up to 2.5 hours post injection of α_ν_β_3_-targeted nanoparticles allows quantification of contrast agent uptake. The temporal evolution of the nanoparticle concentration in the tissue and the blood was used to determine the pharmacokinetic profiles of targeted vs nontargeted particles. Reprinted with permission from Neubauer, et al [49].

CMR of the aortic wall revealed that the targeted nanoparticle agent generated at least two-fold higher signal enhancement at 0.5 and 1 hour post injection than the nontargeted nanoparticles. In both groups, the signal enhancement steadily increased up to 2.5 hours post injection, reflecting the relatively slow transfer of the contrast agent from the blood pool compartment into the angiogenic capillary network where interaction with the α_ν_β_3_-integrin occurs (Figure 1). Both the targeted and nontargeted formulations reached their maximum signal enhancement between 2.5 and 8 hours post injection. The targeted group returned to its baseline value within 12.5 hours post injection. Fitting both the blood clearance and tissue uptake data with a three compartment model showed no significant differences in k_12_, k_21_, v_1_, or k_31 _between the targeted and nontargeted groups. However, k_13 _(representing transfer from the blood into the aortic wall) and k_e _(elimination from the blood pool) were significantly higher for the targeted formulation. While k_13 _represents the passive transfer from the blood into the third compartment in a standard three compartment model, the nanoparticles are not just passively transferred but also actively binding within the third compartment. These two effects can not be differentiated with this model and combine together, resulting in an apparent increase in k_13 _for the targeted nanoparticles compared to the nontargeted formulation.

These pharmacokinetic parameters were used to calculate the area under the curve (AUC), a traditional index of availability. The local tissue concentration of targeted nanoparticles was doubled compared to nontargeted particles (1.64 vs 0.84 nmol Gd^3+^/g tissue/h, respectively) even though their AUCs in the blood were very similar (3.28 vs 3.60 nmol Gd^3+^/mL blood/h). The maximum amount of gadolinium that reached the targeted site relative to the total dose was 0.18% for nontargeted particles and 0.38% for targeted nanoparticles. These results demonstrate that quantitative CMR can be used to noninvasively track binding of a molecularly targeted contrast agent, which has previously only been accomplished with nuclear and optical imaging [52,53]. The primary advantage of this approach is that tissue concentrations of the contrast agent can be calculated noninvasively without the need to collect tissue samples. This allows repeated measurements of the uptake and washout of the agent in the same animal during disease progression or under the influence of a therapeutic intervention. One limitation of this technique, however, is that blood samples spiked with the contrast agent were used to convert the MRI signal enhancement to the tissue nanoparticle concentration. Although the particles are exposed to the blood stream, the image intensity of a voxel also depends upon the tissue relaxation times and water content, which may decrease the accuracy of the particle concentration measurements.

This study demonstrates that pharmacodynamic models can be used to describe the in vivo binding characteristics of site-targeted nanoparticle agents. The lipophilic gadolinium chelate used in this nanoparticle formulation can be envisioned as a surrogate marker for any drugs that might be incorporated into the lipid layer, such as doxorubicin, paclitaxel, or fumagillin [54-56]. Highly lipophilic drugs, such as fumagillin, have very low disassociation rates from the nanoparticles, typically less than 10% of the total dose [56], allowing MRI to provide a reasonable estimate of the drug delivered to tissue. Standard methods for pharmacodynamic modeling rely on blood pool measurements of the drug concentration without considering the accumulation in the target tissue [57,58]. Noninvasive imaging, however, could serially monitor the actual concentration of the drug in the end organ [59], and lead to more accurate modeling of therapeutic response. This feature could become increasingly important as specifically targeted agents are developed, and the clinical need arises to monitor local concentrations of the drug beyond the blood pool.

### Monitoring Endothelial Dysfunction

Quantification of contrast agent uptake has also been utilized in clinical research to map the extent and severity of tissue disruption following ischemic injury. Serial contrast-enhanced cardiac magnetic resonance (CE-CMR) was used to characterize inflammation in the coronary vessel wall of patients after acute myocardial infarction (AMI) [60]. Inflammation plays a key role in the development of atherosclerosis [61] and is closely linked to plaque rupture, the underlying cause of myocardial infarctions and strokes [62]. Plaque vulnerability is determined more by the plaque composition and amount of inflammation rather than the degree of luminal narrowing [63]. Clinical CMR contrast agents nonspecifically distribute throughout the extracellular space, but the amount of leakage out of the capillary bed is dependent upon the permeability and the surface area of the vasculature. Inflammation causes increased vascular permeability, resulting in increased extravasation of the contrast agent. CE-CMR has been used to characterize acute inflammation within the vessel wall in giant cell arteritis and Takayasu's arteritis [64,65]. CE-CMR of the coronary artery wall was performed in 10 patients with AMI 6 days and 3 months after coronary intervention and in 9 volunteers without coronary artery disease [60]. All subjects were imaged on a 1.5T scanner 30-40 minutes after an IV bolus of 0.2 mmol/kg of Gd-DTPA. Contrast enhancement within the coronary wall was calculated based on the contrast-to-noise ratio (CNR) between the image intensity of the coronary wall and the blood signal in the aorta. Noise was measured in a region of interest placed outside the chest wall.

Six days after AMI, the image enhancement in the coronary wall averaged 7.8, which was significantly higher than the enhancement observed in normal subjects, 5.3. Coronary vessel enhancement was highly correlated with the angiographic severity of lumen narrowing. Signal enhancement in the stenotic coronary artery segments, defined as greater than 25% luminal narrowing via x-ray angiography, was significantly higher (CNR = 10.9) than the enhancement measured in the nonstenotic segments (CNR = 6.4). Three months after AMI, the average CNR in the coronary artery wall was 6.5, a significant decrease compared to the value 6 days after AMI. This reduction in average CNR was caused by a decrease in the CNR from the stenotic vessels, CNR = 6.8, while the CNR in the angiographically normal segments did not change between the acute and chronic phase of infarction. The spatial extent of enhanced segments, vessels displaying CNR values above 9.7, decreased from 70% at six days to 25% at three months post AMI. Mirroring the results from CE-CMR, the levels of a general inflammatory marker, C-reactive protein, were significantly higher at six days post AMI compared to the three month timepoint, 2.6 vs. 0.8 mg/dl, respectively. The observed image enhancement pattern during the post-infarction period may be associated with transient inflammation or edema in the pathologically altered coronary vessel wall. Serial CE-CMR could be used to quantify the spatial extent and intensity of coronary inflammation in patients after AMI. Further studies will be required to determine the utility of this approach to predict clinical events or monitor the response to therapeutic interventions. Some therapies designed to increase angiogenesis in the ischemic regions may also significantly increase vascular permeability and dramatically increase CE-CMR image enhancement. Under these conditions, CE-CMR may not accurately reflect local inflammation, but rather the combined physiological effects of both the disease as well as the therapy.

### Drug Delivery

**Figure 2:**
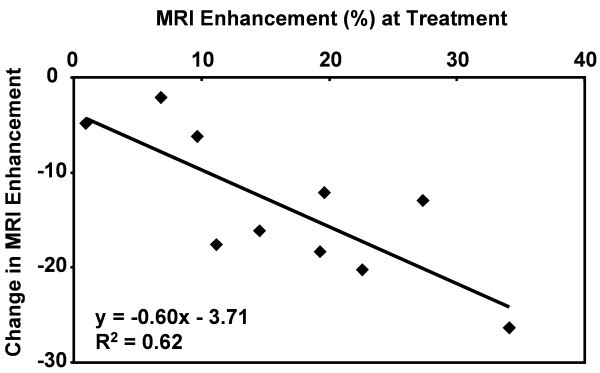
**Quantitative molecular imaging can predict response to anti-angiogenic therapy**. CMR molecular imaging of atherosclerotic rabbits treated with α_ν_β_3_-targeted nanoparticles carrying an anti-angiogenic drug, fumagillin, demonstrated signal enhancement in the aortic wall. Follow-up imaging with α_ν_β_3_-targeted nanoparticles was performed 7 days later to assess residual angiogenic activity in the vessel. Quantitation of enhancement at the time of treatment was related to the amount of drug delivered to the growing atherosclerotic plaques and correlated to the change in signal 7 days after treatment. Sections of the abdominal aorta with the highest signal enhancement at the time of α_ν_β_3_-targeted fumagillin nanoparticle treatment showed the greatest reduction in α_ν_β_3_-integrin expression assessed 1 week later. Reprinted with permission from Winter, et al [56].

Combining a molecularly targeted imaging agent with a therapeutic drug could provide a range of benefits in the clinical management of patients with cardiovascular disease. The imaging agent would allow confirmation and quantification of local drug delivery, and enable personalization of treatment protocols based on the pharmacokinetics in the target tissue. In addition, specific targeting of the agent could improve uptake and retention of the drug at the sites of disease while lowering the exposure to other susceptible organs and reducing the occurrence of side effects. The acute therapeutic response to an anti-angiogenic drug was studied in atherosclerotic rabbits treated with α_ν_β_3_-targeted paramagnetic nanoparticles containing the drug fumagillin [56]. CMR was performed on a 1.5 T clinical scanner to estimate drug deposition in the aortic wall. One week later, the level of neovascular α_ν_β_3_-integrin expression was assessed using α_ν_β_3_-targeted paramagnetic nanoparticles without fumagillin. Aortic areas displaying high CMR enhancement at the time of treatment had the largest subsequent reduction in α_ν_β_3_-targeted CMR signal 1 week later (Figure 2), suggesting that combining imaging with therapy may not only confirm and quantify the local delivery of chemotherapeutics but may also provide early predictions of the subsequent treatment effects.

**Figure 3:**
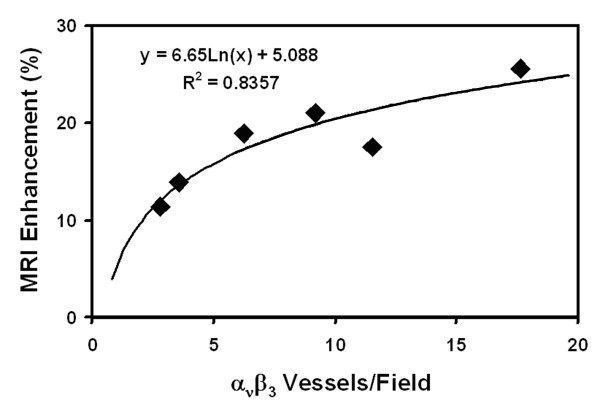
**CMR image enhancement with α_ν_β_3_-targeted nanoparticles correlates to the density of angiogenic microvessels**. CMR molecular imaging of angiogenesis with α_ν_β_3_-targeted paramagnetic nanoparticles was directly related to histological measurement of microvessel density in atherosclerotic rabbits. The number of microvessels expressing both α_ν_β_3_-integrin and platelet/endothelial cell adhesion molecule (PECAM), a general vascular marker, were counted in aortic sections. The microvessel density was correlated in a logarithmic fashion (R^2 ^= 0.84) to the CMR signal enhancement observed after injection of α_ν_β_3_-targeted particles. Reprinted with permission from Winter, et al [55].

In addition to the acute response to therapy, CMR molecular imaging could provide serial monitoring of the end organ effects with the ultimate goal of optimizing drug treatment regiments and customizing patient protocols. The anti-angiogenic effect of atorvastatin with and without targeted delivery of fumagillin was serially monitored for eight weeks by MRI in atherosclerotic rabbits [55]. Atorvastatin was dosed continuously via incorporation into the feed, while fumagillin treatment was provided once every four weeks. Rabbits were imaged on a clinical 1.5T scanner before and three hours post injection of α_ν_β_3_-targeted nanoparticles. Histology revealed that the image enhancement was strongly correlated to the microvascular density in the aortic wall in a logarithmic fashion (Figure 3). During eight weeks of study, atorvastatin treatment did not reduce α_ν_β_3_-integrin expression in the aortic wall. Fumagillin treatment, on the other hand, resulted in a transient (2-3 week) reduction in image enhancement, indicating successful anti-angiogenic treatment. Combining the fumagillin and atorvastatin treatments yielded a persistent decrease in the CMR signal, suggesting that chronic statin treatment could be used to prolong the effects of discrete doses of an anti-angiogenic agent. An effective and sustained anti-angiogenic treatment could stabilize atherosclerotic plaques by decreasing intramural hemorrhage.

Although these studies demonstrate effective drug delivery via targeted nanoparticles and noninvasive monitoring of the therapeutic effect via CMR molecular imaging, there are a number of limitations with the animal model. The atherosclerotic rabbits displayed expanded vasa vasorum and adventitial angiogenesis, but they did not exhibit the pathological signatures of vulnerable plaques, such as intraplaque hemorrhage, thin fibrous caps or plaque rupture. As a result, the ultimate therapeutic effect of anti-angiogenic drugs on the most clinically important atherosclerotic lesions cannot be determined in this animal model. Evaluating these techniques in other animal models, such as dogs or pigs, using more advanced atherosclerotic triggers, such as vascular balloon injury, de-endothelialation or cuffing, will be needed before clinical translation can be considered.

## Quantitative T1/T2 mapping

**Figure 4:**
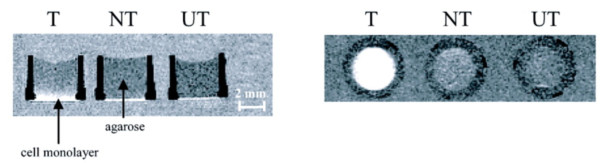
**Molecular imaging of tissue factor expression on cultured smooth muscle cells**. Cell cultures were incubated with tissue factor targeted nanoparticles (T), nontargeted nanoparticles (NT) or no nanoparticles (UT). Left: Spin-echo images of smooth muscle cell monolayers acquired at 1.5T reveal image enhancement for the well treated with targeted particles, but not the wells receiving nontargeted or no nanoparticles. Right: A maximum intensity projection through the 3 D stack of T1-weighted images acquired parallel to the cell monolayers demonstrates the sensitivity of this targeting method for detecting labeled cells. Reprinted with permission from Morawski, et al [74].

Rather than relying on changes in image intensity to quantitate contrast agent uptake, a more accurate method requires calculating relaxation times before and after contrast agent injection. The difference between relaxation times before and after contrast agent injection can be used to directly calculate the tissue concentrations of the agent [66,67]. The binding of paramagnetic nanoparticles targeted to tissue factor expressed on cultured smooth muscle cells was calculated based on changes in T1 relaxation times [66]. This agent carried 94,000 gadolinium chelates per particle, generating longitudinal relaxivities (relative to the concentration of nanoparticles) of 1,690,000 (s*mM)^-1 ^at 1.5T and 910,000 (s*mM)^-1 ^at 4.7T. This extremely high relaxivity results in a minimum detection limit, defined as the concentration of particles required to generate a contrast to noise ratio equal to 5, for this agent of 113 pM, which is within the range of the biological abundance of many important biomarkers. To demonstrate the sensitivity of this high-relaxivity agent to biomarker expression, the nanoparticles were targeted to tissue factor expressed on cultured smooth muscle cells (Figure 4) [66]. Tissue factor is a transmembrane glycoprotein with a prominent role in a number of important biological processes, such as angiogenesis, thrombosis, cell signaling, hemostasis and mitogenesis [68]. Smooth muscle cells comprise the media layer of arteries and are involved in vascular repair following injury and the progression of atherosclerosis.

Cultured smooth muscle cells were exposed to tissue factor targeted nanoparticles or nontargeted nanoparticles. The T1, T2 and proton density of the cell layers were quantified on a 1.5T clinical scanner with a mixed spin echo and inversion recovery sequence. The mixed-scanning protocol combines multiecho spin echo and inversion recovery acquisitions, generating a series of images with different T1 and T2 weightings [50]. The T1 and T2 relaxation times and proton density are calculated from the mixed images based on ratios of the signal intensities and linear least squared fitting. Modeling of the MRI signal enhancement predicted that targeted nanoparticles reached a concentration of 468 pM, while the nontargeted particles reached a concentration of only 88 pM. The modeling estimates were validated by gas chromatography of the samples, revealing a particle concentration of 530 pM for the targeted nanoparticles and 111 pM for the nontargeted nanoparticles.

### Imaging Tissue pH

Some CMR contrast agents have been specifically designed to report on the local physiological conditions, such as temperature, pH or oxygenation. In these cases, the changes in relaxation times are not used to calculate contrast agent concentrations, but rather the physiological parameter of interest. A paramagnetic chelate, Gd-DOTA-4AmP^5-^, was designed to report pH based on changes in the proton exchange kinetics between the bulk water and the metal coordinated water due to protonation of the phosphonates on the side-arms of the chelate [69]. However, a fundamental limitation of any such agent is that the MRI enhancement relies on the local concentration of the agent as well as the tissue pH. To account for the concentration dependence, a non-pH sensitive agent was used to map the wash in and wash out kinetics of the molecule.

For both the pH-sensitive and the pH-insensitive agents, the MRI signal enhancement varied linearly with concentration up to 4 mM. The relaxivity of Gd-DOTP^5- ^was 3.0 1/(mM*s), while the relaxivity of Gd-DOTA-4AmP^5- ^varied from 3.2 to 4.5 1/(mM*s) over a pH range from 5.75 to 8.0. The pH of mouse kidneys was mapped using a 4.7T research scanner. A series of spin echo images were acquired every 40 seconds to track the MRI enhancement for 1 hour post injection. To obtain an accurate measurement of the wash in and wash out kinetics for the pH-sensitive and pH-insensitive agents, Gd-DOTP^5- ^was injected and imaged for 1 hour, followed by injection of Gd-DOTA-4AmP^5- ^and imaging for 1 hour, followed by injection of another dose of Gd-DOTP^5- ^and 1 hour of imaging. In three control mice, the pH of the kidney cortex was 7.3, the medulla had a pH of 7.0, and the calyx-ureter had a pH of 6.3. Immediately after the MRI scans, urine was collected and the pH was measured as 5.9. In addition to the control animals, a group of mice were treated with acetazolamide, a carbonic anhydrase inhibitor that causes systemic metabolic acidosis and alkalinization of the urine due to reduced reabsorption of bicarbonate in the kidney. Acetazolamide raised the pH of the cortex to 7.7, the medulla to 8.0 and the calyx-ureter to 7.5. Urine collected immediately after the CMR scans had a pH of 8.0. The increases in kidney and urine pH compared to the control animals are consistent with the expected mode of action of acetazolamide. Noninvasive mapping of tissue pH could be applied to a number of clinical needs including predicting the therapeutic response of tumors to chemo or radiation therapy [70] or monitoring the progression or treatment of hereditary defects in ion transport in the kidney [71,72]. The need for two contrast agents is a significant limitation of this technique. Any variation in the dose, distribution or elimination of the agent due to changes in animal physiology or the experimental conditions would dramatically reduce the accuracy of the pH measurements.

A further modification of this pH-sensitive paramagnetic contrast agent, Gd-DOTA-4AmP^5-^, has allowed the tissue concentration to be determined directly by PET imaging [73]. An ^18^F tag was coupled to one of the pendant arms of the original chelate structure. Using a hybrid MR-PET imaging system, the concentration of the agent and the change in relaxation times can be determined, providing a means to calculate pH. This method, however, requires instrumentation that can perform both MR and PET imaging, which is not widely available. In addition, the resolution of PET systems is significantly lower than CMR scanners, resulting in much larger voxels for the concentration map compared to the relaxation time map. How these differences effect the final spatial resolution for pH imaging will need to be determined.

## Direct Quantitation of CMR Contrast Agents

While changes in the proton signal intensity or relaxation times can be used to measure contrast agent uptake or tissue physiology, these methods are indirect measures of the contrast agent. An alternative method is direct detection of a signal originating from the agent itself, much as PET, SPECT and optical imaging directly map a nuclear or fluorescent tag incorporated onto the agent. There are a number of important nuclei that are visible by CMR techniques, such as ^19^F, ^23^Na, ^31^P, and ^13^C, however, the sensitivity of these elements tend to be quite low. Typical clinical MRI utilizes the proton (^1^H) signal, which represents a concentration of 110 M and a relative MRI sensitivity of 1.0. In comparison, the relative sensitivities of ^19^F, ^23^Na, ^31^P, and ^13^C are 0.83, 0.093, 0.066 and 0.016, respectively. Furthermore, the concentrations of these elements tend to be very low in biological samples, typically less than 10 mM, lowering their sensitivity by orders of magnitude compared to proton.

### ^19^F CMR of Perfluorocarbon Nanoparticles

**Figure 5:**
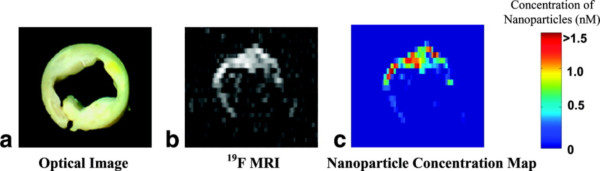
**Direct quantitation of contrast agent binding utilizing ^19^F CMR and fibrin-targeted PFC nanoparticles**. (a) Optical image ex vivo of a 5-mm cross section of a human carotid endarterectomy sample. This section showed moderate luminal narrowing as well as several atherosclerotic lesions. (b) A ^19^F projection image acquired at 4.7 T through the entire carotid artery sample shows high signal along the lumen due to nanoparticles bound to fibrin. (c) Concentration map of bound nanoparticles in the carotid sample. Reprinted with permission from Morawski, et al [74].

Direct detection of ^19^F has been explored in a number of research studies because it has a relatively high sensitivity, 83% compared to ^1^H, and there is virtually no native background signal. Thus, fluorinated contrast agents can provide a definitive and quantitative CMR signature. For example, ^19^F CMR of fibrin-targeted perfluorocarbon (PFC) nanoparticles could be used to map the formation of thrombi on ruptured atherosclerotic plaques and quantify the extent of ruptures in the fibrous cap. Human carotid endarterectomy samples were treated with fibrin-targeted paramagnetic nanoparticles and imaged at 4.7 T [74]. ^1^H CMR showed high levels of signal enhancement along the luminal surface due to nanoparticle binding to fibrin deposits. A ^19^F projection image of the artery, acquired in less than five minutes, displayed an asymmetric distribution of nanoparticles around the vessel wall corroborating the ^1^H signal enhancement. Spectroscopic quantification of nanoparticle binding allowed calibration of the ^19^F CMR signal intensity. Co-registration of the quantitative nanoparticle map with the ^1^H image permitted visualization of both anatomical and pathological information in a single image (Figure 5). Combining information from ^1^H and ^19^F CMR could allow prediction of subsequent occlusion or distal embolization from unstable or disrupted plaques, and aid clinical decision-making for acute invasive intervention vs. pharmaceutical therapies.

Further experiments performed on a 1.5 T clinical CMR system used rapid steady-state imaging to independently image and quantify two different populations of fibrin-targeted nanoparticles, perfluorooctylbromide (PFOB) or perfluoro-15-crown-5-ether (CE), based on their unique spectral signatures [75]. Both imaging and spectroscopy could distinguish nanoparticles containing either PFOB or CE as the core material. The signal to noise for PFOB was lower than CE (10 vs. 25, respectively), presumably due to the single CE peak (20 equivalent fluorine atoms) compared to the multiple PFOB peaks (17 fluorine atoms distributed over 5 peaks). A clear linear relationship between the ^19^F signal intensity and perfluorocarbon concentration was demonstrated for both PFOB and CE using both imaging and spectroscopy. From this demonstration on fibrin clots, it follows that multiple perfluorocarbon nanoparticle agents could be used to target different epitopes and achieve a noninvasive analogy to immunohistochemistry. For example, simultaneous quantification of angiogenesis in the vessel wall and fibrin deposition on the plaque cap could be used to evaluate the pathophysiological stage of an obstructive lesion.

**Figure 6:**
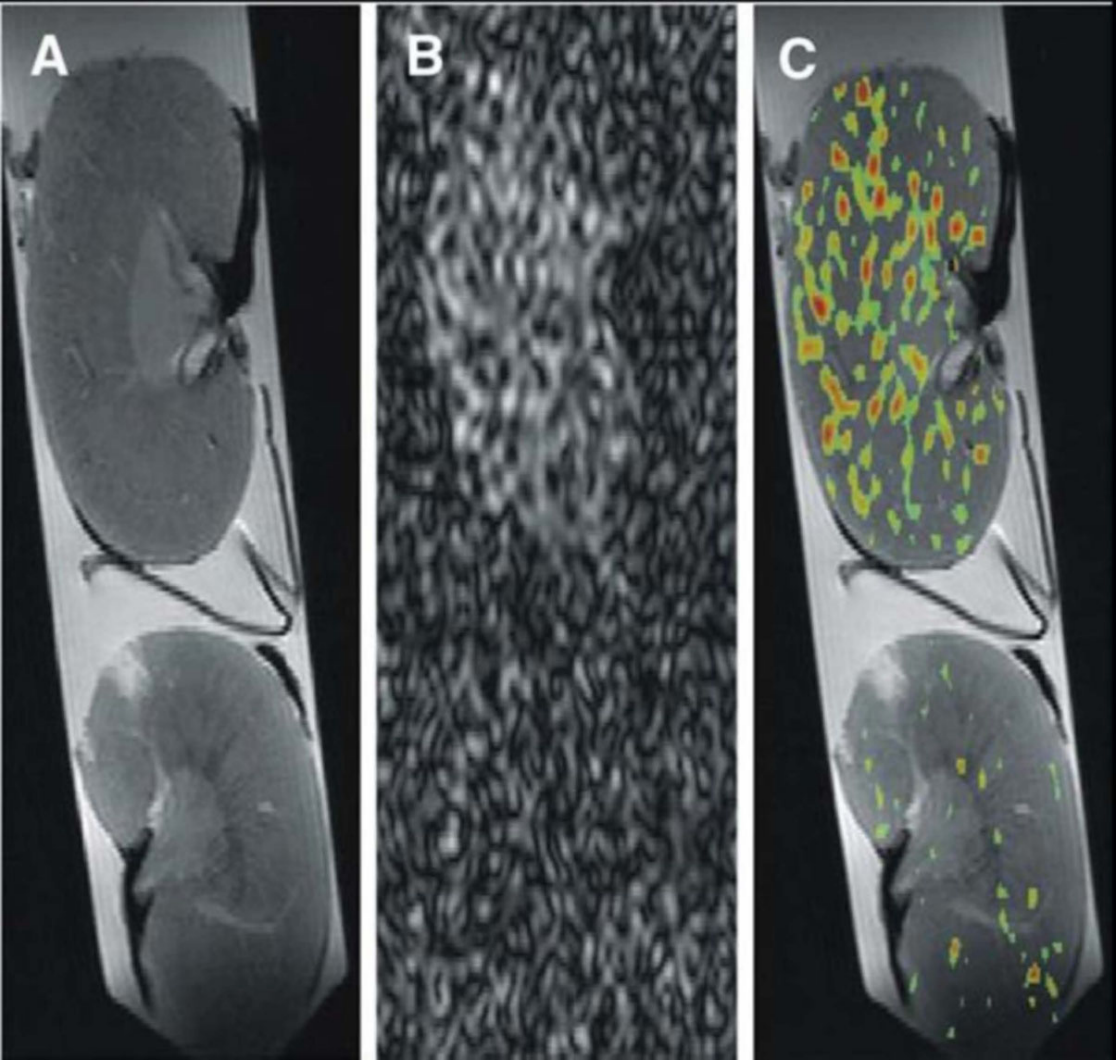
**Mapping tissue uptake of VCAM-1 targeted PFC nanoparticles with ^19^F CMR**. Multinuclear imaging of kidneys from atherosclerotic ApoE^-/- ^(top) and wild-type control (bottom) mice imaged at 11.7T. (A) Proton MR of kidney anatomy. (B) ^19^F CMR for direct detection of VCAM-1-targeted PFC nanoparticles. (C) A composite ^1^H/^19^F image allows precise overlay of molecular biomarker of inflammation and anatomical detail. Reprinted with permission from Southworth, et al [76].

Tissue inflammation is another biological marker of unstable atherosclerotic plaques, inducing the release of cytokines and upregulating the production of proteins like vascular cell adhesion molecule-1 (VCAM-1). PFC nanoparticles targeted to VCAM-1 were injected into genetically engineered ApoE^-/- ^mice, which mimic the clinical progression of atherosclerosis, to map inflammation [76]. These mice display focal inflammation and macrophage infiltration in the kidneys [77]. To definitively identify nanoparticle binding in the kidneys, ^19^F CMR was performed on a 11.7 T research scanner 2 hours after injection of VCAM-1 targeted PFC nanoparticles (Figure 6). The ^19^F signal arising from the PFC core provided an unambiguous marker of particle accumulation, without the innate signal variations that can confound typical ^1^H signal enhancement with traditional paramagnetic or superparamagnetic CMR contrast agents. VCAM-1-targeted nanoparticles accumulated in ApoE^-/- ^kidneys to a greater extent than non-targeted nanoparticles (3.7 billion particles per gram of tissue vs. 0.9 billion particles/gram). The uptake of targeted nanoparticles was also higher in the kidneys of ApoE^-/- ^mice compared to non-ApoE^-/- ^controls (3.7 vs. 1.6 billion particles/gram). Control animals also displayed no significant difference in the uptake of targeted versus non-targeted nanoparticles (1.6 vs. 1.5 billion particles/gram).

Typically, targeted CMR contrast agents generate image enhancement as a result of both nonspecific blood pool signal as well as the specific binding of the agent to the biomarker of interest. Separating out these two contributions can be very difficult to achieve in vivo. One method to suppress the nonspecific signal utilizes diffusion weighted ^19^F spectroscopy to null the signal arising from moving particles, representing the unbound fraction in the blood pool, while retaining the signal from stationary particles that are specifically bound to the target epitope [78]. A genetically engineered mouse model of squamous cell cancer, derived by incorporating human papilloma virus into the mouse genome [79], was studied with a 11.7 T research scanner. Transgenic and age matched control mice were injected with 1 ml/kg α_ν_β_3_-targeted PFC nanoparticles (corresponding to a PFC dose of 0.2 ml/kg) and scanned 90 minutes later to assess angiogenesis in these precancerous lesions. Both the transgenic and control mice displayed decreased ^19^F signal with increasing b-values. However, 60-100% of the ^19^F signal remained at b-values near 60,000 s/mm^2 ^in the transgenic animals, while no detectable ^19^F signal was observed in control mice at b-values of 1500 s/mm^2^. The calculated apparent diffusion coefficient (ADC) of PFC nanoparticles was 33.1 m^2^/s in the transgenic mice, significantly lower than the 19,563 m^2^/s ADC in the controls.

Angiogenesis is also a prominent feature in the progression of aortic valve stenosis. Cholesterol-fed rabbits develop aortic valve sclerosis, characterized by gross thickening, macrophage infiltration, calcification, and eventual bone formation that mimics the clinical presentation of the disease [80-83]. Cholesterol-fed rabbits underwent ^19^F CMR after injection of α_ν_β_3_-targeted nanoparticles to quantify angiogenesis in the aortic valve leaflets [84]. The cholesterol feeding caused gross thickening of the aortic valves accompanied by extensive foam cell infiltration, non-calcified bone deposition, activation of myofibroblasts, abnormal microvascular proliferation and upregulation of α_ν_β_3_-integrin expression. None of these abnormalities were observed in the normal valve tissue from control animals. Rabbits received IV injections of 2.2 ml/kg α_ν_β_3_-targeted nanoparticles, nontargeted nanoparticles or in vivo competitive inhibition of α_ν_β_3_-integrin binding via pretreatment with α_ν_β_3_-targeted safflower oil nanoparticles. Two hours after nanoparticle injection, the aortic valve leaflets were excised for ^19^F MR spectroscopy at 11.7T.

**Figure 7:**
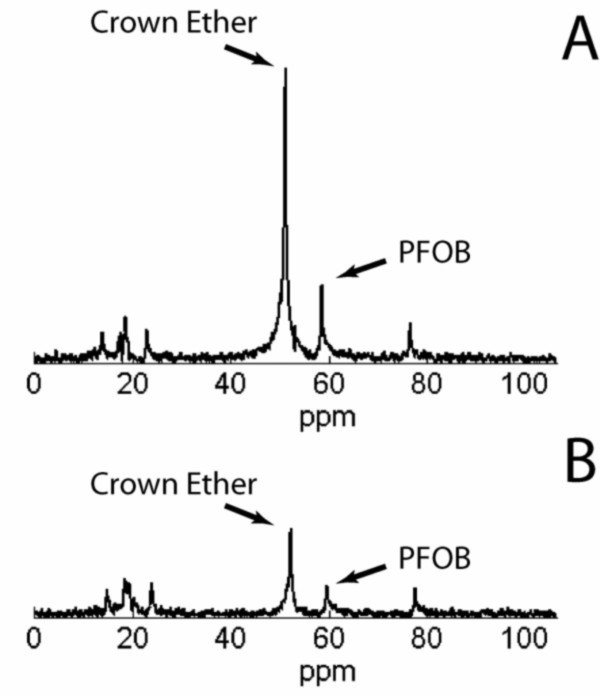
**Quantitative ^19^F spectroscopy of angiogenesis in aortic valve disease**. The ^19^F signal was utilized to quantify binding of nanoparticles to the valve leaflets from (A) a rabbit treated with α_ν_β_3_-targeted nanoparticles and (B) a rabbit treated with untargeted nanoparticles. Both nanoparticle formulations consisted of a crown ether core, which generates a single peak. The PFOB peaks originated from a reference standard utilized for quantification. Nanoparticle binding in the rabbit treated with targeted particles was much higher (Crown Ether/PFOB = 4.6) than the rabbit treated with nontargeted particles (Crown Ether/PFOB = 2.2). Reprinted with permission from Waters, et al [84].

**Figure 8:**
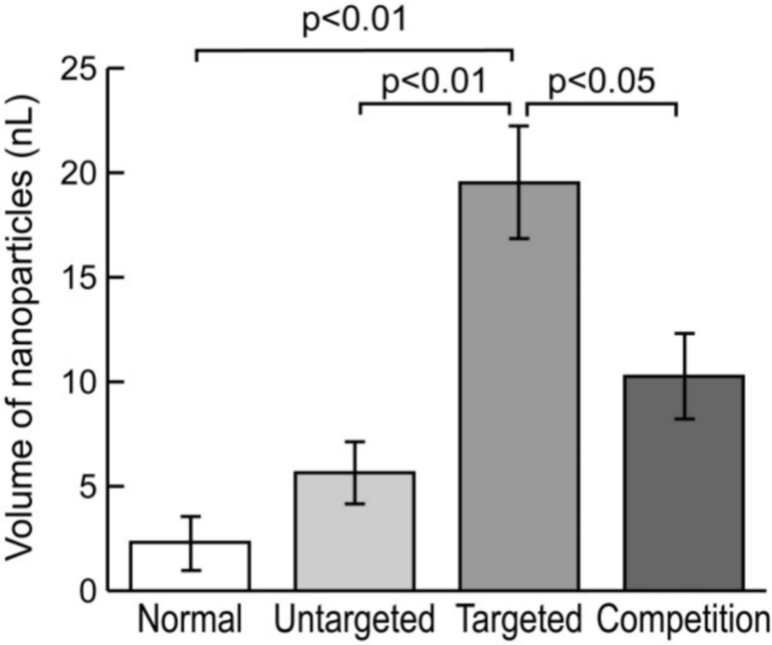
**Quantitative comparison of nanoparticle binding in valve leaflets**. The volume of nanoparticles (in nanoliters) bound to the valves was calculated from the ^19^F signal. The valves treated with α_ν_β_3_-targeted particles displayed three times higher signal compared to the nontargeted formulation and twice the signal of valves with competitive inhibition of α_ν_β_3_-integrin binding. Minimal nanoparticle deposition occurred in non-atherosclerotic animals treated with targeted nanoparticles due to the lack of angiogenesis in the valve. Reprinted with permission from Waters, et al [84].

The crown ether (CE) signal arising from the nanoparticle contrast agent was readily detected and distinguished from a perfluorooctylbromide (PFOB) quantification reference based on the chemical shifts of these perfluorocarbon species (Figure 7), allowing quantification of the total volume of bound nanoparticles (Figure 8). The volume of targeted nanoparticles bound to the valves was 19.5 nL, which was more than three times higher than the amount of nontargeted nanoparticles (5.6 nL). Competitive inhibition of α_ν_β_3_-integrin binding reduced the amount of nanoparticles in the valves by about half (10.3 nL). Valves from healthy rabbits treated with targeted nanoparticles contained almost nine times fewer nanoparticles (2.3 nL) than the valves from cholesterol-fed rabbits. These techniques may be useful for assessing atherosclerotic components of preclinical aortic valve disease in patients and could assist in defining efficacy of medical therapies. The sensitivity of this approach for molecular detection of sparse quantities of inflammatory epitopes in very thin structures at high field strengths establishes a basis for future efforts to develop localized spectroscopic methods at clinical field strengths that could be useful for detecting disease and monitoring therapies.

The ability of ^19^F CMR to directly detect and quantitate the binding of specifically targeted nanoparticles is a significant advantage over the use of paramagnetic or superparamagnetic contrast agents, which are only visible based on their effects on the water signal. However, a lingering disadvantage of these techniques is the limited sensitivity of CMR to any signal other than the bulk water. These studies usually overcome this inherent limitation by using some combination of high magnetic field strength, employing MR spectroscopy, and/or very long scan times.

### Mapping Tissue Oxygenation

As with proton imaging, ^19^F contrast agents have been developed for mapping tissue physiology, most notably oxygenation. Oxygen is essential for tissue viability and without an adequate supply, cellular dysfunction and death rapidly occurs. Noninvasive monitoring of tissue oxygenation could be utilized for diagnostic and therapeutic applications in a range of common diseases, including myocardial ischemia, cancer, stroke and peripheral vascular disease. The most common CMR technique to monitor tissue oxygenation is Blood Oxygen Level Dependant (BOLD) imaging. BOLD imaging is sensitive to the ratio of oxyhemoglobin and deoxyhemoglobin and can be utilized for high spatial and temporal mapping of the dynamic changes in brain oxygenation during functional CMR studies [85]. However, the BOLD CMR signal is not quantitatively related to tissue oxygenation, because it depends strongly on a number of other factors including blood volumes, blood flow, hematocrit and pH [86]. Other CMR oximetry techniques use contrast agents that are sensitive to the local oxygen tension (pO_2_). A number of these agents are based on perfluorocarbon molecules, which display a linear dependence of the ^19^F spin lattice relaxation rate on pO_2 _[87]. In order to accurately measure pO2, the T1 relaxation time of the fluorinated compound must be quantified in order to avoid the influence of contrast agent concentration, T2 and other factors on the CMR signal. Molecular oxygen is paramagnetic and the solubility of oxygen in perfluorocarbons is three to ten times higher than in water [88,89]. Oximetry based on ^19^F CMR capitalizes on a number of strengths: it is a spin 1/2 nucleus, the sensitivity is approximately 83% compared to ^1^H, it is 100% abundant, and endogenous fluorine in biological samples only occurs at very low levels and is typically undetectable because of very short T2 relaxation times. Due to the lack of a background ^19^F signal in the body, PFC agents can be definitively identified and quantified by CMR.

PFCs generating multiple spectroscopic resonances can be used provide multiple estimates of pO_2 _or to measure pO_2 _and another physiological parameter, such as temperature, that affects the ^19^F R1 value by solving simultaneous equations [90]. For imaging, however, the multiple ^19^F resonances can generate chemical shift artifacts or reduce the overall signal-to-noise [91,92]. PFCs with a single resonance, such as perfluoro-15-crown-5-ether or hexafluorobenzene offer high pO_2 _sensitivity and minimal temperature sensitivity [93-95]. For example, hexafluorobenzene has been investigated for mapping tumor oxygenation during hyperoxic interventions using echo planar imaging (EPI) to maximize the temporal resolution [95].

### Quantification of Metabolic Flux

Another important physiological marker of disease is metabolic flux rates. For example, the creatine kinase (CK) reaction is responsible for replenishing adenosine triphosphate (ATP) by using phosphocreatine (PCr). Quantitative ^31^P spectroscopy studies have shown significant reductions in cardiac PCr and ATP concentrations in MI patients compared to healthy controls [96]. However, these measurements do not distinguish between cell death, reduced substrate availability or impaired enzyme activity. MR spectroscopy can be used to measure the rate of metabolic reactions by tracing the exchange of saturated spins from one molecule to another. The pseudo-first-order CK rate constant, k, reflects the intracellular CK reaction kinetics and is independent of myocyte number, while CK flux is defined as the product of [PCr] and k. The value of k can be interpreted as the fraction of the PCr pool used to create ATP via the CK reaction each second, which is a measure of intracellular metabolic function. Therefore, k depends only on the surviving cells that contribute to the ^31^P MRS signal and is not confounded by myocyte loss. On the other hand, reduced myocardial CK flux can be due to a loss of total enzyme activity, altered intracellular substrate levels, or allosteric modifications of the enzyme.

In a clinical study of myocardial infarction patients, the CK kinetics were measured noninvasively using ^31^P spectroscopy. The tissue concentrations of ATP and PCR were measured and CK kinetics (k and CK flux) were measured by magnetization transfer on a 1.5T clinical scanner [97]. Myocardial [ATP] and [PCr] were 39% to 44% lower in MI patients compared to healthy controls, however the myocardial CK rate constant, k, was normal in these patients. As a result of the lower tissue PCr levels, the CK flux was reduced by 50% in the MI patient population. These results demonstrate that ATP loss following MI is a direct result of PCr depletion, most likely due to myocyte loss. The maintenance of normal k values indicates that intracellular CK metabolism is maintained in the surviving myocytes. These results reinforce the use of therapies for MI patients that combat substrate loss or reduce energy demand, rather than those that increase workload in the surviving tissue. For example, beta blockers are routinely prescribed for MI patients because they reduce the heart rate and myocardial oxygen consumption. Using similar ^31^P spectroscopy techniques, a 50% reduction in CK flux has been measured in patients with non-ischemic dilated cardiomyopathy and mild-to-moderate chronic heart failure (CHF) [98] and a 65% decrease in CK flux has been reported in patients with pressure-overload left ventricular hypertrophy and CHF [99].

Although this study demonstrates ^31^P CMR on clinical patients, there are a number of technical hurdles that limit the wide-spread use of this method in the clinic, including the instrumentation required, limited resolution and long scan times. ^31^P spectroscopy requires specialty instrumentation that is not found on standard clinical CMR scanners, such as dedicated cardiac ^31^P coils and data processing packages. Also, the ^31^P data was localized in only one dimension, yielding a relatively poor resolution of roughly 6.5 by 6.5 by 1 cm. This would limit the application to patients with relatively large and well defined MIs. Furthermore, completion of the spectroscopy exam took about 70 minutes. As a result, the other CMR exams, including cine, tagging and delayed myocardial enhancement, were performed during a separate imaging session.

## Conclusions

Quantitative CMR molecular imaging encompasses a wide range of developing technologies, such as site-targeted contrast agents, drug delivery vehicles, activatable CMR probes and direct mapping of tissue metabolites. As demonstrated by the research studies cited in this review, the clinical application of these techniques could offer far reaching benefits to patient populations, including early detection of therapeutic response, localizing ruptured atherosclerotic plaques, stratifying patients based on biochemical disease markers, tissue-specific drug delivery, confirmation and quantification of end-organ drug uptake, and noninvasive monitoring of disease recurrence. In particular, molecular imaging with PFC nanoparticle agents has demonstrated a number of applications in animal models of cardiovascular disease. The ability to combine ^1^H and ^19^F CMR offers anatomical localization as well as definitive and quantitative mapping of nanoparticle uptake. Utilizing the nanoparticle platform has proven to be highly flexible, enabling selection of various contrast mechanisms, targeting ligands or therapeutic drugs based on the requirements of the specific application. Eventually, such agents may play a leading role in reducing the human burden of cardiovascular disease, by providing early diagnosis, noninvasive monitoring and effective therapy with reduced side effects.

## Competing interests

SAW and GML are founders and minority stock holders in Kereos, Inc. SAW receives research support from Philips Healthcare. PMW and SDC have no competing interests to declare.

## Authors' contributions

All authors contributed to the scope and outline of the manuscript. PMW wrote the final draft. All authors read and approved the final manuscript.
